# Haptic Shared Control Framework with Interaction Force Constraint Based on Control Barrier Function for Teleoperation

**DOI:** 10.3390/s25020405

**Published:** 2025-01-11

**Authors:** Wenlei Qin, Haoran Yi, Zhibin Fan, Jie Zhao

**Affiliations:** State Key Laboratory of Robotics and System, Harbin Institute of Technology, Harbin 150001, China; 18b908096@stu.hit.edu.cn (W.Q.); 23b908051@stu.hit.edu.cn (Z.F.)

**Keywords:** shared control, force constraint, Control Barrier Functions, teleoperation, medical robots and systems

## Abstract

Current teleoperated robotic systems for retinal surgery cannot effectively control subtle tool-to-tissue interaction forces. This limitation may lead to patient injury caused by the surgeon’s mistakes. To improve the safety of retinal surgery, this paper proposes a haptic shared control framework for teleoperation based on a force-constrained supervisory controller. The supervisory controller leverages Control Barrier Functions (CBFs) and the interaction model to modify teleoperated inputs when they are deemed unsafe. This method ensures that the interaction forces at the slave robot’s end-effector remain within the safe range without the robot’s dynamic model and the safety margin. Additionally, the master robot provides haptic feedback to enhance the surgeon’s situational awareness during surgery, reducing the risk of misjudgment. Finally, simulated membrane peeling experiments are conducted in a controlled intraocular surgical environment using a teleoperated robotic system controlled by a non-expert. The experimental results demonstrate that the proposed control framework significantly reduces the rate of force constraint violation.

## 1. Introduction

Retinal diseases are a major cause of blindness [1]. Treating these conditions often requires retinal microsurgery. Common surgical procedures include peeling the epiretinal membrane (ERM), which has an average thickness of 60 μm [2], and the internal limiting membrane (ILM), which averages 2 μm in thickness [3]. Thus, retinal microsurgery demands exceptional depth perception and precise operations, presenting significant challenges for the surgeon [4]. Furthermore, the forces exerted between the tool tip and the membrane throughout the peeling process are below the perception threshold of most surgeons [5]. Excessive forces can easily cause unintended damage to the delicate retina, which lacks regenerative capacity, potentially leading to long-term functional impairment [6].

Teleoperation is one of the key methods to address these challenges in retinal microsurgery. Teleoperated robotic systems employ motion scaling to effectively suppress hand tremor. This greatly improves surgical positioning accuracy [7]. Edwards et al. first reported the clinical application of teleoperated robots for ERM and ILM peeling to repair macular holes [8]. The Intraocular Robotic Intervention Surgical System (IRISS) is a novel surgical system that combines automation and teleoperation with augmented reality [9]. A hybrid parallel-serial teleoperated device developed by the Technical University of Munich overcomes the surgeon’s hand tremor to achieve better dexterous movement [10]. Johns Hopkins University developed a teleoperation control mode to reduce scleral damage [11]. This mode incorporates a scleral force control algorithm on the Steady-Hand Eye Robot (SHER). It uses Fiber Bragg Grating (FBG) sensors to measure scleral force in real-time. However, the aforementioned teleoperated robotic systems for retinal surgery have notable shortcomings. They lack control over tool tip forces and lack haptic feedback. These issues increase the risk of surgeon misjudgment and may harm the patient.

Teleoperated robotic systems are required to constrain the forces exerted on the environment to meet safety requirements. To achieve this, various control methods have been proposed. Ruwanthika et al. [12] proposed a constrained bilateral control method capable of maintaining safe force limits in a remote environment when excessive forces are applied to the master system. In addition to constraining excessive forces on the master robot, Nicola et al. proposed a distributed Nonlinear Model Predictive Control (NMPC) framework to directly limit the interaction forces between the slave robot and the environment [13]. Beyond teleoperation research, significant advancements have been made in the field of robotic interaction control. For example, Liang et al. introduced a novel Force-Constrained Control Barrier Function (FCCBF) and developed a force controller based on FCCBF and a quadratic programming (QP) problem [14]. This method minimally modifies the impedance controller, enabling the manipulator’s interaction process to maintain the desired compliance while ensuring that contact forces remain within an allowable force range. However, these methods rely on the robot’s dynamic model, which poses two main challenges in practical applications. First, interaction forces in retinal surgery are typically around 20 mN [15]. These small forces are difficult to estimate through the robot’s dynamics. Second, retinal surgery also requires a Remote Center of Motion (RCM) mechanism, whose complex dynamics are difficult to model accurately. Hence, retinal surgery robots commonly rely on kinematic control schemes, using low-level joint controllers to meet the stringent requirements for accuracy and stability [8,9,10,11]. For these reasons, there is an urgent need for teleoperation methods that do not rely on the robot dynamics model and have force constraint capabilities.

In retinal robotic surgery, researchers have focused on force constraint studies for cooperative-control and handheld systems. For example, Uneri et al. used a proportional force controller to regulate the tool tip forces of the cooperative-control robot [16]. To improve the stability of force control, Wells et al. implemented impedance control to constrain tool tip forces of the handheld device (Micron) [17]. Building on this, Ebrahimi et al. proposed an adaptive force/position hybrid control method successfully applied to the SHER for constraining scleral forces [18]. However, these force control methods typically suffer from overshooting. To reduce overshoot risk, researchers typically define a force control activation value below the safety threshold. The force controller activates when the actual force reaches the activation value. The force range between the activation value and the safety threshold is called the “safety margin” [18]. Although the safety margin improves surgical safety to some extent, it also introduces limitations. Activating the force controller prematurely reduces the stable force range available within the safe force range. This may impair the accessibility of surgical tools to the target surgical sites. Thus, a force-constrained method that eliminates reliance on the safety margin is urgently needed.

To improve the safety of retinal surgery, we propose a haptic shared control framework for teleoperation based on a force-constrained supervisory controller. The framework uses CBF and the interaction model to impose strict constraints on the slave robot’s velocity commands without the robot’s dynamics model. By constructing and solving a constrained QP problem, the force-constrained supervisory controller minimally modifies the user’s commands when they are deemed unsafe. This ensures that the interaction forces at the tool tip remain within the safe range without introducing a safety margin. Furthermore, the tool tip forces and the deviation between the supervisory controller’s commands and the user’s commands are amplified and fed back to the master robot to enhance the surgeon’s situational awareness. This feedback mechanism enhances system transparency and helps the surgeon better perceive interaction force changes in the surgical environment.

The characteristics and contributions of our work are as follows:

(1) A haptic shared control framework based on a force-constrained supervisory controller for teleoperation is proposed. This framework modifies teleoperated inputs when they are deemed unsafe to ensure that the interaction forces remain within the safe range without introducing the robot’s dynamic model and the safety margin.
(2) Artificial membrane peeling experiments are conducted using a master–slave robotic system to validate the feasibility and effectiveness of the proposed framework.

This paper is organized as follows: Section 2 describes the master–slave robotic system used in this study. The mathematical model of the master–slave robots is presented in Section 3, along with the control design. The simulation results are discussed in detail in Section 4. The experimental results are discussed in detail in Section 5. Section 6 presents the further discussion. Finally, the conclusions are drawn in Section 7.

## 2. Experimental Setup

**Figure 1:**
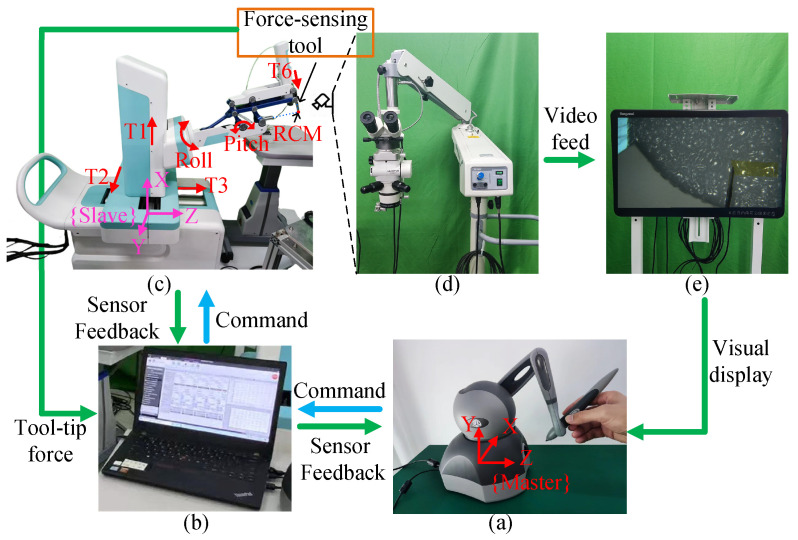
Experimental setup. (**a**) Master robot: receives user operation commands. (**b**) Interface computer: collects force data, generates, and transmits motion commands to the slave robot and control signals to the master robot. (**c**) Slave robot with force-sensing tool: performs surgical operations. (**d**) Microscope: provides high-resolution magnified images of the surgical site. (**e**) Monitor: displays the surgical procedure for the surgeon.

The master–slave robotic system used in the experiment is shown in Figure 1. The master robot is a haptic device manufactured by 3D Systems(Rock Hill, SC, USA). It communicates with the interface computer via USB at 1 kHz. The slave robot, developed by the Harbin Institute of Technology [19], is a 6-degree-of-freedom (DOF) velocity-controlled robot with four translational movements (T1, T2, T3, and T6) and two rotational movements (Roll and Pitch), as shown in Figure 1c. The intersection of the Roll and T6 axis forms the RCM. Before surgery, the RCM must be aligned with the scleral incision through the movement of T1–T3 axes, thus constraining the tool movement relative to the eye at the scleral entry point. Communication between the interface computer and the slave robot is achieved via the Modbus TCP protocol at a frequency of 30 Hz, while the slave robot control algorithm runs at a sampling frequency of 500 Hz. The visualization of the intraocular surgical process is provided by an electronic microscope (Olympus Co., Tokyo, Japan) positioned above the patient’s eye and displayed to the operator on a 26-inch monitor(IKEGAMI TSUSHINKI Co., Tokyo, Japan).

**Figure 2:**
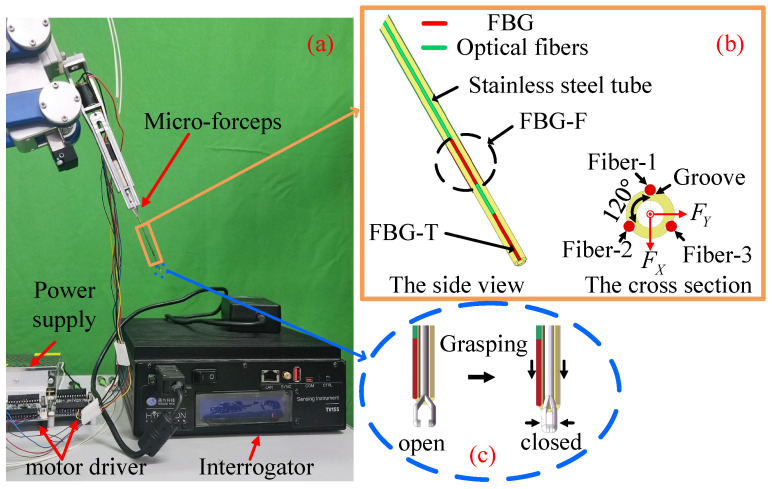
Overview of the force-sensing subsystems. (**a**) System components, including the micro-forceps, power supply, motor driver, and interrogator. (**b**) Diagram of the tubular tool shaft with the FBG sensors. (**c**) Diagram of the clamping mechanism of the micro-forceps.

The force-sensing subsystem includes the micro-forceps, power supply, motor driver, and interrogator. The micro-forceps is mounted as the end-effector of the slave robot to measure interaction forces and clamp the membrane, as shown in Figure 2a. Three optical fibers are arranged at 120° intervals along the stainless steel tube of the micro-forceps, as shown in Figure 2b. Each of the two fibers contain a single 5 mm FBG sensor, while the third fiber contains two 5 mm FBG sensors spaced 4 mm apart. The FBG sensors are categorized by function as FBG-T and FBG-F. FBG-T, located near the fiber end, is used for temperature compensation. FBG-F includes the remaining three FBG sensors and measures three-dimensional forces. The optical sensing interrogator(Beijing Tongwei Co., Beijing, China) measures the wavelength of light reflected by the FBG sensors and transmits these data to the computer at a frequency of 1 kHz. The computer then calculates force data based on these measurements. The micro-forceps also has a 0.4 mm hollow channel, enabling integration with a grasper. As shown in Figure 2c, the steel tube moves forward along the axis of the grasper to close the grasper through compression. The force calibration method for the force-sensing tool is described in detail in [20].

## 3. Mathematical Models and Haptic Shared Control Framework

**Figure 3:**
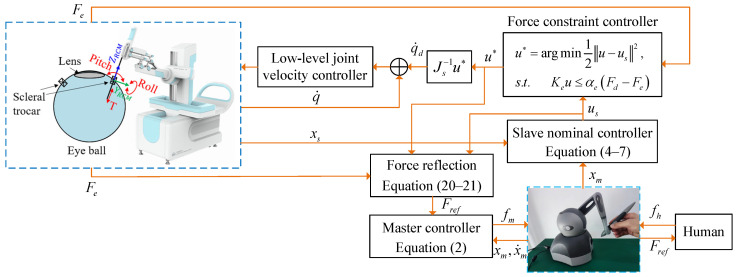
Block diagram of the haptic shared control architecture.

To help the surgeon to focus on surgery and prevent tissue injury from excessive interaction forces, this paper proposes a haptic shared control framework for teleoperation. The control block diagram is shown in Figure 3. The operator uses the master robot to control the slave robot. The force constraint controller on the slave robot adjusts the commands from the operator when the interaction forces are about to exceed the safe thresholds, maintaining safe force levels. The master robot also provides haptic feedback to indicate the interaction forces and the extent to which the user’s commands deviate from the safe commands.

### 3.1. Master Robot

The master robot used in this paper can be modeled as

(1) $$M\left(x_{m}\right){\ddot{x}}_{m}+C\left(x_{m},{\dot{x}}_{m}\right){\dot{x}}_{m}+G\left(x_{m}\right)=f_{m}+f_{h},$$

(2) $$f_{m}=F_{ref}-B_{m}{\dot{x}}_{m}+\tilde{G}\left(x_{m}\right),$$

where $x_{m}\in R^{3}$ represents the tip position of the master robot, $M\left(x_{m}\right)\in R^{3\times 3}$ denotes the inertia matrix, $C\left(x_{m},{\dot{x}}_{m}\right)\in R^{3\times 3}$ is the Coriolis/centrifugal terms, and $G\left(x_{m}\right)\in R^{3}$ represents the gravitational terms. The terms $f_{m},f_{h}\in R^{3}$ denote the controller of the master robot and force applied by the operator, respectively. $B_{m}\in R^{3\times 3}$ is a positive definite diagonal matrix representing the virtual damping, which stabilizes the haptic device. $\tilde{G}\left(x_{m}\right)\in R^{3}$ is a gravity compensation term, while $F_{ref}\in R^{3}$ denotes the reference force for haptic feedback. The reference force guides the operator to move in the direction that adheres to the constraints.

### 3.2. Slave Robot

The slave robot is a velocity-controlled robot. Only three degrees of freedom (Roll, Pitch, and T6) associated with the RCM mechanism are utilized during the surgical operation. The desired joint velocities are calculated using the robot’s inverse velocity kinematics:

(3) $${\dot{q}}_{s}=J^{†}u_{s},$$

where $u_{s}$ is the end-effector velocity and $J^{†}\in R^{3\times 3}$ represents the pseudo inverse of Jacobian matrix of the slave robot. The vector ${\dot{q}}_{s}\in R^{3}$ is sent to the low-level joint velocity PID controller.

The slave robot is instructed to track the trajectory from the master robot using the following feedforward and feedback control scheme:

(4) us=x˙ds+κ(xds−xs),

(5) xds=λRmsxm+xcoms,

(6) x˙ds=λRmsx^˙m=λRmsζ2,

where $x_{s}\in R^{3}$ represents the position of the slave robot, xds∈R3 is the compensated position of the master robot described in the base frame of the slave robot, $\kappa \in R^{+}$ is the feedback control gain, and x˙ds∈R3 is the feedforward velocity. $\lambda \in R^{+}$ is a position scaling factor to suppress the operator tremors and enable precise manipulation. Rms∈R3×3 is the rotation matrix that transforms the master robot frame into the slave robot frame, and xcoms∈R3 is the offset of the master–slave robots during connection or reconnection. $\zeta_{2}\in R^{3}$ is the estimated velocity of the master robot, generated by the following differentiator [21]:

(7) $$\begin{array}{c} {\dot{\zeta }}_{1}=\zeta_{2}+l\alpha_{1}\left(x_{m}-\zeta_{1}\right)\\ {\dot{\zeta }}_{2}=l^{2}\alpha_{0}\left(x_{m}-\zeta_{1}\right) \end{array},$$

where l>0 is the filter gain, and $\alpha_{0},\alpha_{1}>0$ are selected to ensure the characteristic equation $p\left(s\right)=s^{2}+\alpha_{1}s+\alpha_{0}$ is Hurwitz. This master–slave coupling method enables the operator to control the slave robot by manipulating the master robot.

### 3.3. Force-Constrained Supervisory Controller

Ophthalmic surgical robots generally operate at lower speeds to minimize the risk of soft-tissue damage. As a result, the slave robot can be modeled as a single integrator, with the following state-space representation:

(8) $$\begin{array}{c} {\dot{x}}_{s}=f\left(x_{s}\right)+g\left(x_{s}\right)u,\\ f\left(x\right)=\left[\begin{array}{c} 0\\ 0\\ 0 \end{array}\right],g\left(x\right)=\left[\begin{array}{ccc} 1 & 0 & 0\\ 0 & 1 & 0\\ 0 & 0 & 1 \end{array}\right] \end{array},$$

where $u={\left[\begin{array}{ccc} u_{x} & u_{y} & u_{z} \end{array}\right]}^{T}\in R^{3}$ is the input, corresponding to a velocity vector.

Given that the control of the slave robot is decoupled in XYZ directions, a continuously differentiable function is defined for each DoF:

(9) $$h_{i}\left(x_{s}\right)=F_{di}-F_{ei}\left(x_{s}\right):R^{3}\to R,i\in \left\{X,Y,Z\right\},$$

where $F_{di}\in R$ denotes the maximum constraint force. $F_{ei}\left(x_{s}\right)\in R$ represents the tool-to-tissue interaction force. In order for the interaction force to satisfy $\left|F_{ei}\right|\leq F_{di}$, the safe set $C\subset R^{3}$ of the function $h_{i}\left(x_{s}\right)$ should meet the following conditions:

(10) $$\left\{\begin{array}{c} C=\left\{x_{s}\in R^{3}:h_{i}\left(x_{s}\right)\geq 0\right\},\\ \partial C=\left\{x_{s}\in R^{3}:h_{i}\left(x_{s}\right)=0\right\},\\ Int\left(C\right)=\left\{x_{s}\in R^{3}:h_{i}\left(x_{s}\right)>0\right\}, \end{array}\right.$$

where ∂C represents the boundary of the set, and $Int\left(C\right)$ denotes the interior of the set.

**Theorem** **1.** *For any initial position $x_{s0}\in C$, there is a maximum time interval $\left[0,t_{\max}\right)$. The solution to system (8) satisfies $x_{s}\left(t\right)\in C$ when $u_{i}$ meets the following conditions:*

(11) $${\dot{h}}_{i}\left(x_{s},u_{i}\right)\geq -\alpha_{ei}h_{i}\left(x_{s}\right).$$

In this case, the set C is a forward invariant set, and $h_{i}\left(x_{s}\right)=0$ is called CBF [22]. $\alpha_{ei}$ is a positive convergence factor. ${\dot{h}}_{i}\left(x_{s},u_{i}\right)=L_{f}h_{i}\left(x_{s}\right)+L_{g}h_{i}\left(x_{s}\right)u_{i}$. $L_{f}h_{i}\left(x_{s}\right)=\frac{\partial h_{i}}{\partial x_{s}}\left(x_{s}\right)f\left(x_{s}\right)$, $L_{g}h_{i}\left(x_{s}\right)=\frac{\partial h_{i}}{\partial x_{s}}\left(x_{s}\right)g\left(x_{s}\right)$ are in the form of a standard Lie derivative.

**Proof of Theorem** **1.** Define a function as follows:

(12) $$z\left(t\right)={\dot{h}}_{i}\left(t\right)+\alpha_{ei}h_{i}\left(t\right).$$

${\dot{h}}_{i}\left(t\right)\geq -\alpha_{ei}h_{i}\left(t\right)$ shows that $z\left(t\right)$ is non-negative; then, the solution to the first-order differential Equation (12) is

(13) $$h_{i}\left(t\right)=h_{i}\left(0\right)e^{-\alpha_{ei}t}+\int_{0}^{t}e^{-\alpha_{ei}\left(t-r\right)}z\left(t\right)dr.$$

Since the second term on the right side is non-negative, we can conclude that

(14) $$h_{i}\left(t\right)\geq h_{i}\left(0\right)e^{-\alpha_{e}t}\geq 0.$$

Proof completed. □

Assume that force-displacement model of the slave robot can be approximated by a simple linear spring model, as described in [23]:

(15) $$F_{e}=\left\{\begin{array}{c} K_{e}\left(x_{s}-x_{r}\right),\begin{array}{cc} & x_{s}\geq x_{r} \end{array}\\ 0,\begin{array}{cc} & x_{s}<x_{r} \end{array} \end{array}\right.,$$

where $x_{r}\in R^{3}$ represents the environmental position at which forces begin to act on the slave robot’s end-effector. $K_{e}\in R^{3}$ is a stiffness matrix with positive diagonal entries and zero off-diagonal entries, and $k_{ei}$ denotes the *i* direction diagonal entry of $K_{e}$.

Combining the system model (8) with the interaction model (15) yields that $L_{f}h_{i}\left(x_{s}\right)=0$, $L_{g}h_{i}\left(x_{s}\right)=-k_{ei}$. So, the force controller $u_{i}$ should satisfy the following constraint that ensures $\left|F_{ei}\right|\leq F_{di}$:

(16) $$k_{ei}u_{i}\leq \alpha_{ei}\left(F_{di}-F_{ei}\right).$$

The force constraint (16) needs to adjust the slave nominal controller (4) as little as possible to ensure that the interaction force satisfies $\left|F_{ei}\right|\leq F_{di}$. With this in mind, the following QP problem is formulated to generate the Lipschitz continuous controller $u^{*}\in R^{3}$:

(17) $$\begin{array}{c} u^{*}=\arg\min\frac{1}{2}{∥u-u_{s}∥}^{2},\\ s.t.\begin{array}{cc} & K_{e}u\leq \alpha_{e}\left(F_{d}-F_{e}\right) \end{array} \end{array}$$

which can be solved analytically if the number of active constraints is limited to the number of degrees of freedom, i.e., $\dim u\geq \dim\left[\begin{array}{ccc} h_{X}\left(x_{s}\right) & h_{Y}\left(x_{s}\right) & h_{Z}\left(x_{s}\right) \end{array}\right]$. The constraints in the optimization problem ensure that the slave nominal controller is used in directions where the force constraints are inactive.

**Theorem** **2.** *Consider the slave robot model (8) and the interaction model (15). Under the force supervisory controller (17), all signals in the closed-loop system of the slave robot are bounded. When the CBF constraints (16) are inactive, the slave robot accurately follows the motions of the master robot under the action of $u_{s}$. Once the CBF constraints are activated, the slave robot transitions to force control mode, ensuring $F_{e}$ converges to $F_{d}$.*

**Proof of Theorem** **2.** The Lagrangian function of QP problem (17) is presented as follows.

(18) $$L=\frac{1}{2}{∥u-u_{s}∥}^{2}+\lambda^{T}\left(K_{e}u-\alpha_{e}\left(F_{d}-F_{e}\right)\right),$$

where $\lambda \in R^{3}$ is a Lagrangian multiple. The explicit form of (17) is derived using the Karush–Kuhn–Tucker (KKT) conditions, as shown below.

(19) $$u^{*}=\left\{\begin{array}{c} u_{s},x_{s}\in \Pi_{0}\\ u_{s}+\Delta u,x_{s}\in \Pi_{I} \end{array}\right.,$$

where Δu represents the correction term caused by the force constraint (16). $\Pi_{0}$ and $\Pi_{I}$ represent the sets of inactive and active constraints, respectively. The stability of the closed-loop system is analyzed based on whether $x_{s}$ belongs to $\Pi_{0}$ or $\Pi_{I}$.

Case 1: the slave nominal controller $u_{s}$ satisfies all force constraints. At this point, all force constraints are inactive. The closed-loop motion equation of the slave robot is x˙s=x˙ds+kxds−xs. Define the tracking error e=xds−xs; then, $\dot{e}+ke=0$ holds. For ∀k>0, as t→∞, e→0. Thus, the slave robot can accurately follow the movement of the master robot, and all signals in the closed-loop system are bounded.

Case 2: At least one force constraint is active. To simplify the analysis, we assume that only one force constraint is active. In this case, ${\dot{h}}_{i}=-\alpha_{ei}h_{i}$. The candidate Lyapunov function is $V_{i}=\frac{1}{2}h_{i}^{2}$. The time derivative of $V_{i}$ is ${\dot{V}}_{i}=h_{i}{\dot{h}}_{i}=-\alpha_{ei}h_{i}^{2}=-2\alpha_{ei}V$. Since $\alpha_{ei}>0$, ${\dot{V}}_{i}$ is negative definite, and $h_{i}$ is asymptotically stable as an equilibrium point. Therefore, if one of the force constraints is active, the slave robot transitions from teleoperation mode to force control mode, and the force tracking error exponentially converges to a compact set of zero. According to Equations (3), (8), and (15), the position of the slave robot and the joint angles are bounded.

Proof completed. □

### 3.4. Reference Force

To provide the surgeon with the situational awareness of the slave robot, we define the reference forces $F_{ref}\in R^{3}$ at the master side,

(20) $$F_{ref}=-\beta F_{e}+F_{g},$$

where $\beta \in R^{+}$ is a scaling factor, and the guiding forces $F_{g}\in R^{3}$ represent the deviation between the user command $u_{s}$ and the controller $u^{*}$:

(21) $$F_{g}=k_{g}\left(u^{*}-u_{s}\right),$$

where $k_{g}\in R^{+}$ is a positive constant.

The forces $F_{ref}$ can indicate the interaction forces on the tool tip. When the interaction forces reach the maximum constraint forces, $F_{ref}$ also direct the user to the revised safety instructions.

## 4. Simulation Verification

**Figure 4:**
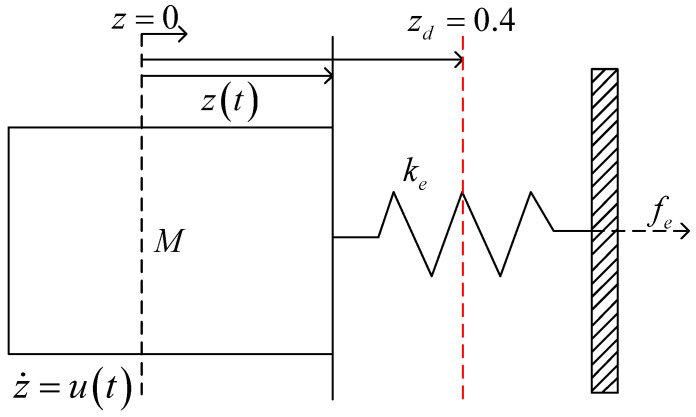
Schematic diagram for the force control of a robot interacting with an environment.

A simplified teleoperation simulation experiment was designed to validate that the proposed method eliminates the need for the safety margin. In the experiment, a 1DOF velocity-controlled robot started at an initial position of 0 and interacted with the environment via the motion controller *u*, as shown in Figure 4. The operator command $z_{d}$ was set to a constant value of 0.4 to simulate a remote operation scenario. The interaction model was described by Equation (8), where the environmental position z=0 m, and the stiffness coefficient $K_{e}=1000$ N/m. The maximum interaction force was limited to 20 N. The proposed method, a force/position hybrid controller, and an impedance controller were tested. Table 1 presents the controller *u* and the corresponding parameters for the three methods. The parameters were selected to maximize the response speed while maintaining approximately the same steady-state error.

**Figure 5:**
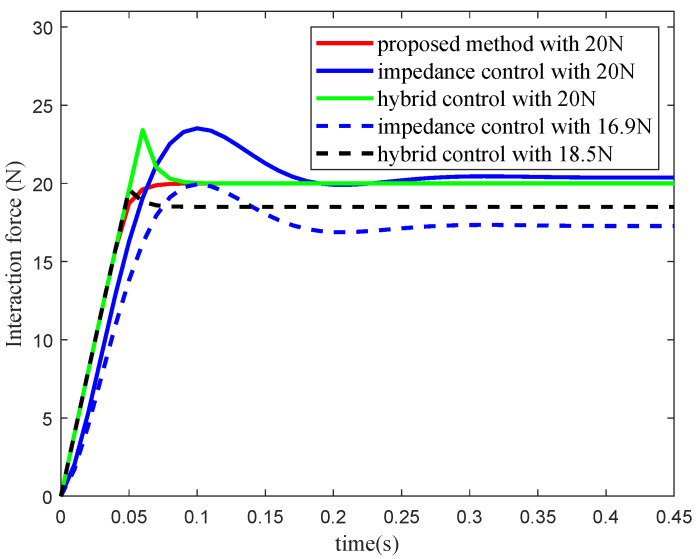
Force curves under different force activation values.

**Figure 6:**
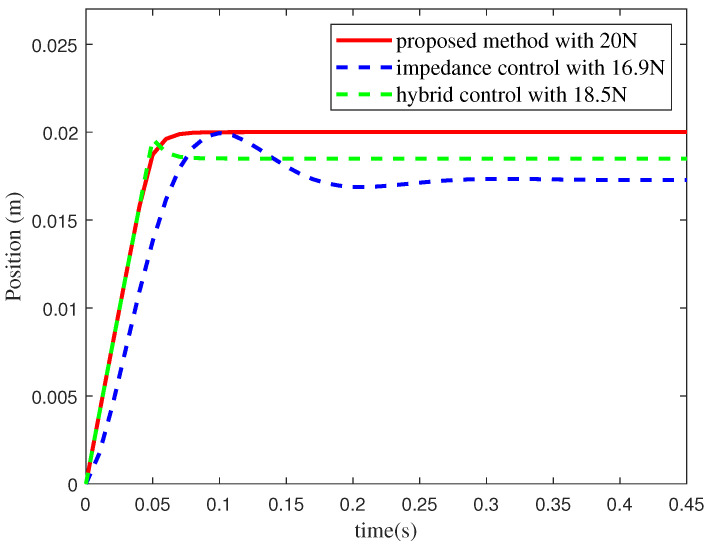
Position curves under different force activation values.

The interaction force curves of the three controllers are shown in Figure 5. When the force activation value fd=20 N is set for the force/position hybrid controller and the impedance controller, it produces interaction forces exceeding 20 N, as shown by the green and blue solid lines in Figure 5. With the force activation values of fd=18.5 N for the force/position hybrid controller and fd=16.9 N for the impedance controller, the interaction forces remain precisely below the maximum limit. This is illustrated by the black and blue dashed lines in Figure 5. However, selecting appropriate activation values requires multiple trials. These results show that the two methods require safety margins of [18.5 N, 20 N] and [16.9 N, 20 N], respectively. In contrast, the proposed method ensures that the interaction force remains within the maximum limit without the safety margin, as shown by the red solid line in Figure 5. Additionally, the proposed method allows the robot to operate freely across the full interaction force range [0, 20 N], whereas the other two methods are restricted to narrower ranges of [0, 18.5 N] and [0, 16.9 N], respectively. This indicates that the proposed method allows the robot to operate freely within a broader force range. Although the safety margin keeps the interaction force within the safe range, it limits the displacement range, as shown in Figure 6. Under the condition that the interaction force does not exceed the safety boundary, the steady-state displacement of the proposed method is 0.02 m, which is greater than 0.0185 m for the force/position hybrid controller and 0.0173 m for the impedance controller.

## 5. Experimental Results

Membrane peeling, including ERM and ILM peeling, is a standard procedure in retinal surgery. Common artificial membranes used for experimental validation include liquid bandage [24], clear bandage [25], and clingwrap [26]. To evaluate whether the proposed method satisfies strict interaction force constraints in teleoperation, a tape peeling experiment was performed to simulate membrane peeling in a controlled intraocular surgical environment.

**Figure 7:**
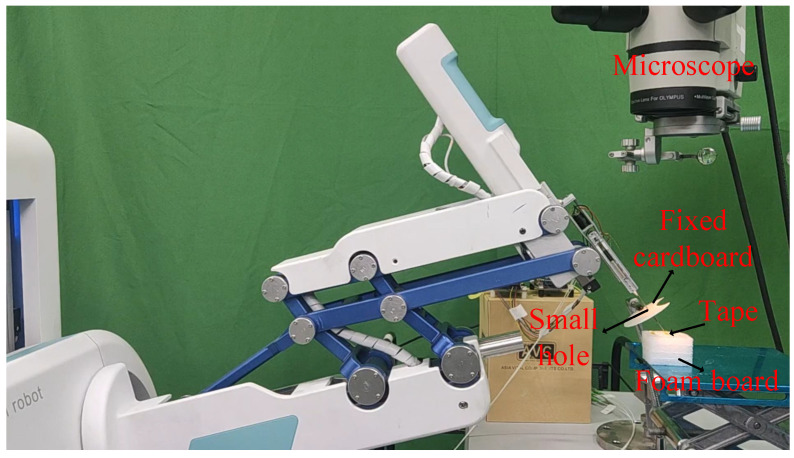
Simulated environment for robot-assisted membrane peeling.

The controlled environment for membrane peeling in retinal surgery is illustrated in Figure 7. A 3 mm diameter hole was created at the center of a fixed circular cardboard to simulate an incision in the sclera. A 5-mm-long piece of yellow tape was affixed to the top surface of a foam block, representing the preretinal membrane and retina, respectively. The task required the user to peel the tape via teleoperation, with the goal of minimizing the interaction forces between the membrane and the forceps as much as possible. The user perceived the peeling process through haptic feedback from the master robot and visual feedback from the monitor. As this study focuses on force constraints during membrane peeling, the RCM of the slave robot was aligned with the cardboard hole, and the forceps clamped one end of the tape before the experiment began.

For comparison, the Telemanipulation Integration Architecture (TIA) from previous work [27] was implemented, as has been reported for use in peeling liquid bandages. Three membrane peeling experiments were conducted using the proposed control method, TIA method, and TIA with haptic feedback for membrane peeling. A novice without prior teleoperation experience was recruited. The novice was chosen because inexperienced operators are more likely to apply excessive force during the operation, facilitating the validation of the proposed force constraint strategy. Before formal data collection, the operator practiced each membrane peeling method for ten minutes per method. Data collected in each experiment included the interaction forces, position of the master robot, position of the slave robot, haptic feedback forces, and time information. Based on previous studies [15,28], the peeling force typically ranges from 8 mN to 45 mN; thus, the maximum constraint force for the peeling process in this study was set to 20 mN. Forces exceeding the range of [−20, 20] mN were considered dangerous, with greater excess indicating higher risk levels.

**Figure 8:**
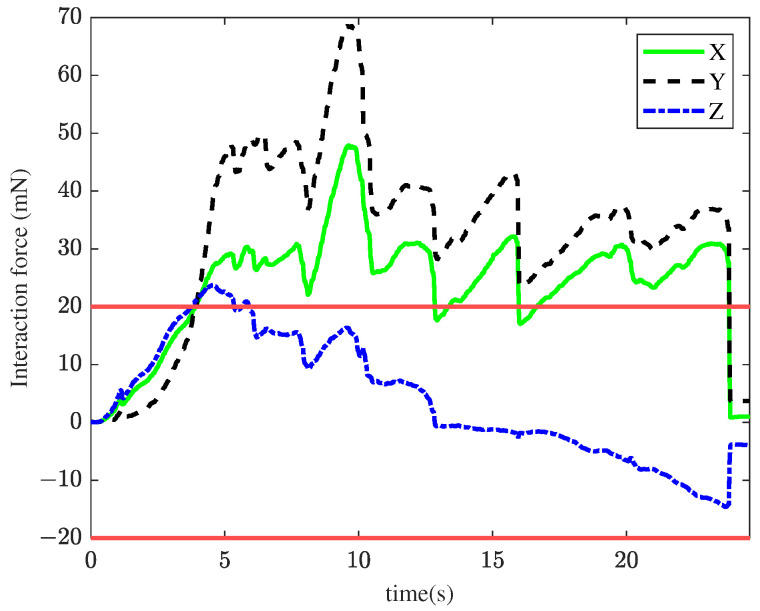
Results of the first experiment: interaction force using the TIA method. The red lines represent the maximum constraint force.

**Figure 9:**
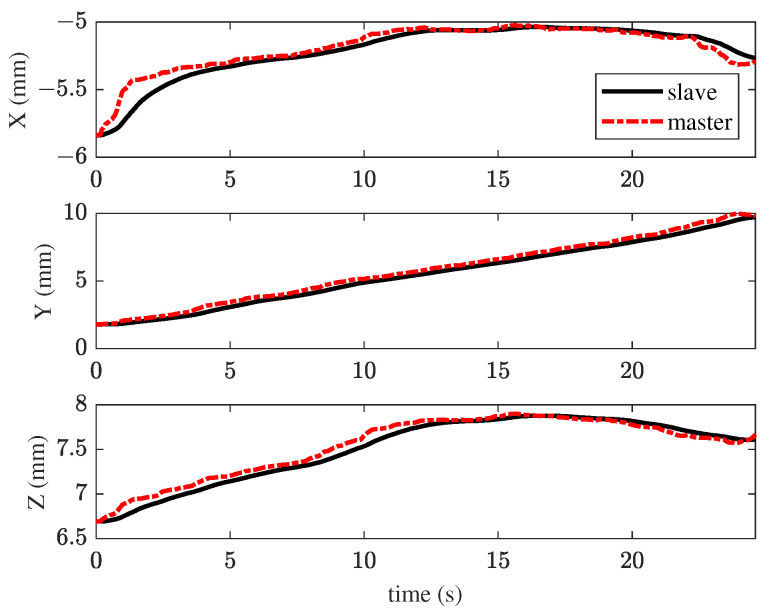
Results of the first experiment: position tracking performance of master–slave robots using the TIA method.

In the first experiment, the TIA method was applied for membrane peeling. The control parameters were set as $k_{t}=0.1$, $\alpha_{t}=0.4$, $v_{\max}=0.01$ m/s. The parameters were selected to maximize the position tracking response speed while maintaining approximately the same tracking error at the slave robot’s end-effector. Figure 8 illustrates the interaction forces at the tool tip during peeling, where the red line represents the maximum constraint force. The forces in the X and Y directions exceed the maximum constraint force for most of the time, with a peak force of 68.6 mN. Such excessive forces are unacceptable in retinal surgery, as they could cause significant harm to the patient. Figure 9 shows the tracking performance between the master and slave robots throughout the peeling process. This indicates that the user’s operation was swift and fluid. Under TIA conditions, the operator relied solely on limited visual information during the procedure. Despite achieving higher task efficiency, it is difficult to effectively reduce interaction forces.

**Figure 10:**
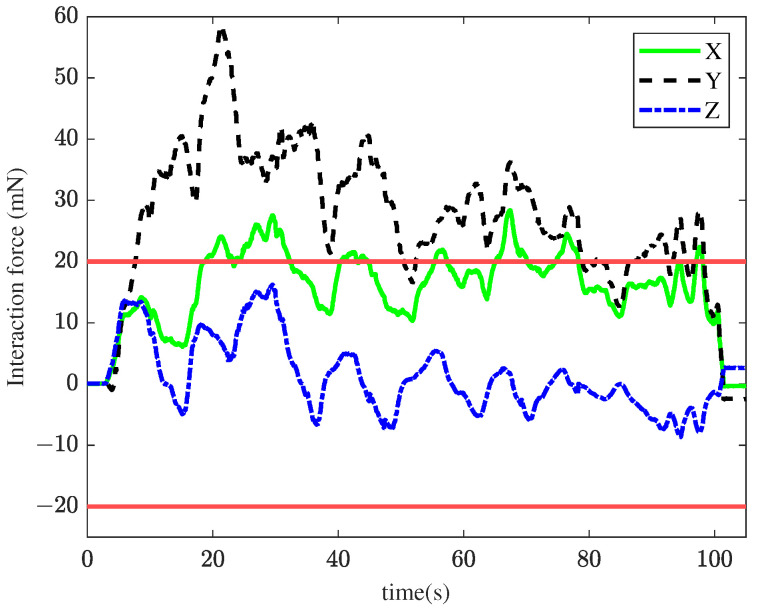
Results of the second experiment: interaction force using the TIA method with haptic feedback. The red lines represent the maximum constraint force.

**Figure 11:**
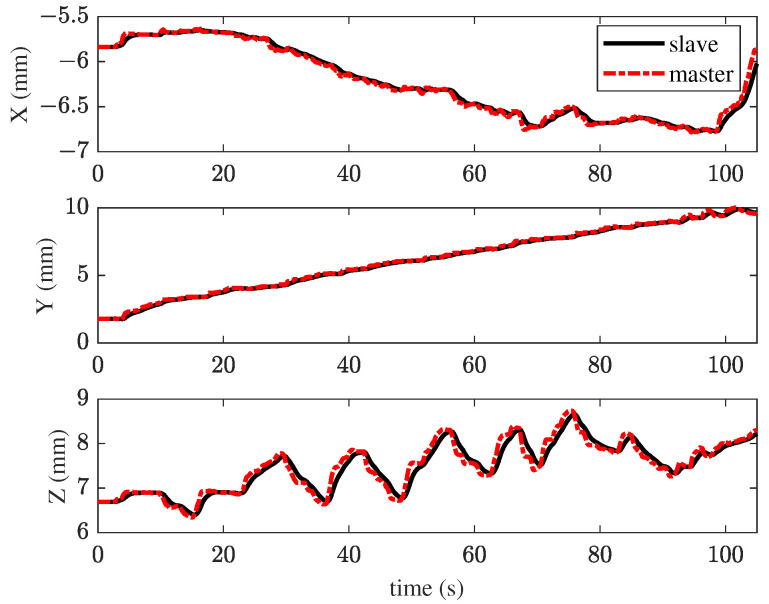
Results of the second experiment: position tracking performance of master–slave robots using the TIA method with haptic feedback.

**Figure 12:**
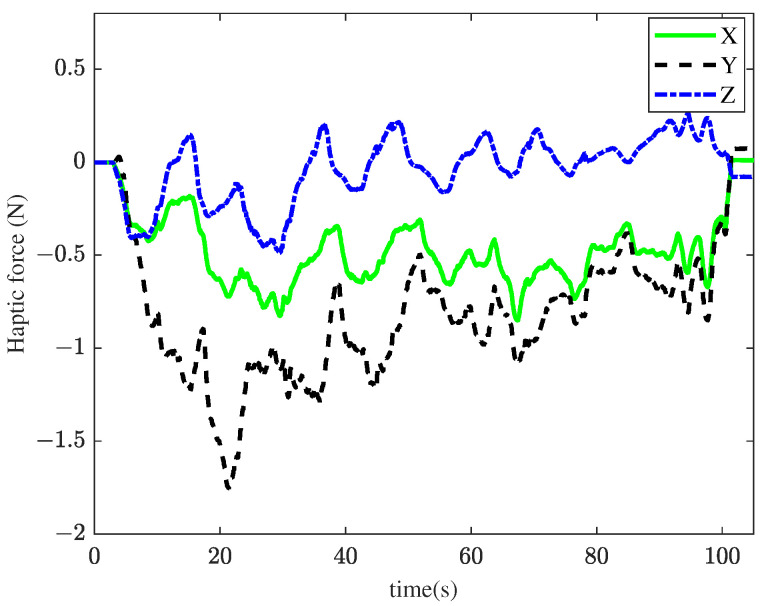
Results of the second experiment: haptic force using the TIA method with haptic feedback.

In the second experiment, the TIA method with haptic feedback was applied to the membrane peeling task. The control parameters from the TIA method were used, with additional interaction force feedback $\beta F_{e}$, where β=0.03. The peeling forces in all directions slightly decreased, as shown in Figure 10. However, in the primary peeling direction (Y), excessive force still occurs, with a maximum value of 58.5 mN. Figure 11 illustrates the tracking performance between the master and slave robots. Figure 12 presents the haptic force generated by the master device during peeling. As shown in Figure 11, the task time significantly increased, indicating that the operator became more cautious. Although task efficiency decreased after introducing haptic feedback, the interaction forces were slightly reduced.

**Figure 13:**
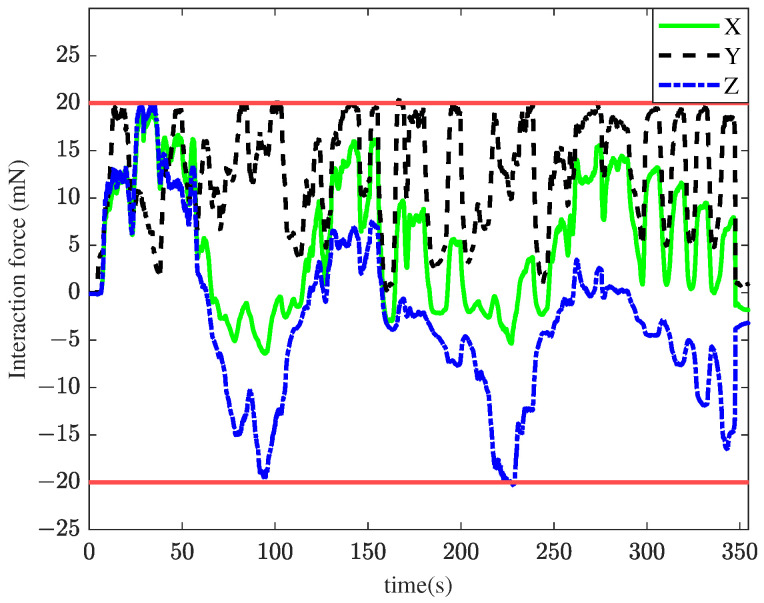
Results of the third experiment: interaction force using the proposed method. The red lines represent the maximum constraint force.

**Figure 14:**
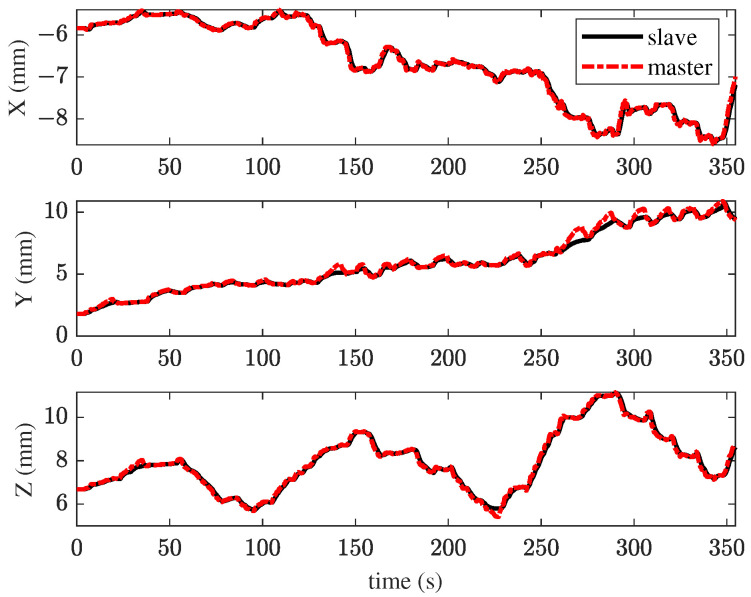
Results of the third experiment: position tracking performance of master–slave robots using the proposed method.

**Figure 15:**
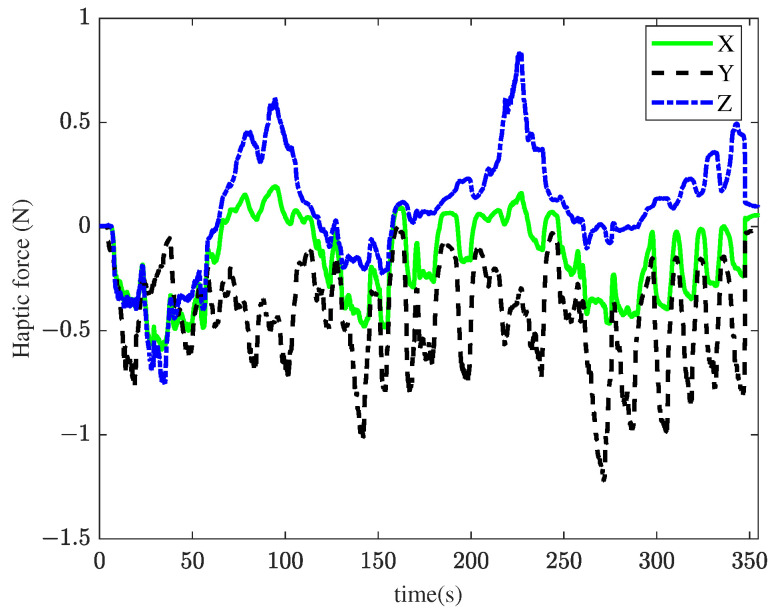
Results of the third experiment: haptic force using the proposed method.

**Figure 16:**
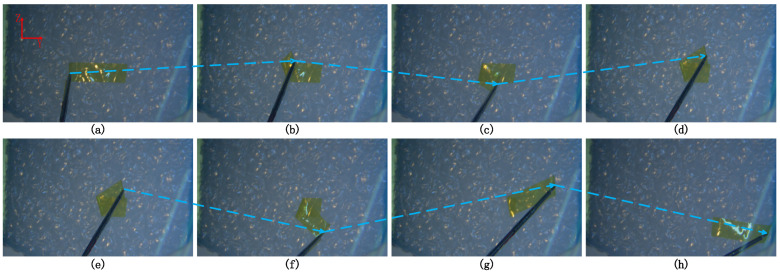
(**a**–**h**) Snapshots of the membrane peeling task using proposed method. The blue dashed arrows show the tool progresses in a folding pattern along the Y-axis.

In the third experiment, the proposed method was applied to the membrane peeling task. The control parameters were set as $B_{m}=3I_{3\times 3}$, κ=1, λ=0.1, $\alpha_{1}=4$, $\alpha_{0}=10$, l=14.28, $K_{e}=500I_{3\times 3}$, $\alpha_{e}=20$, β=0.03, and $k_{g}=0.6$. The qpOASES [29] was used to solve controller (17). Figure 13 illustrates the interaction forces applied to the tool tip, showing that the force remained within the safe range of [−20, 20] mN throughout the task. Figure 14 presents the master–slave tracking performance during the membrane peeling. At *t* = 142 s, 153 s, 271 s, 287 s, 305 s, and 318 s, the Y-coordinate of the slave robot deviated from the master robot. This occurred because as the Y-direction interaction force approached the maximum constraint, the user’s continued movement triggered constraint conditions (Inequality (16)), modifying the user’s nominal commands. Similarly, deviations in the Z-coordinate were observed at *t* = 35 s and 226 s for the same reason. Figure 15 shows force feedback provided by the haptic device during the membrane peeling task. Frequent and alternating increases in haptic forces along the Z and Y directions were observed. This was due to the operator’s continuous attempts to move in the Z direction after feeling immobility in the Y direction. The peeling time for the proposed method was the longest among the three methods. This was because the proposed approach restricts direct movement along the Y-axis to avoid excessive interaction forces. Instead, the tool progresses in a folding pattern along the Y-axis, as shown in Figure 16. This extended the tool’s movement path, resulting in a longer task completion time.

Additionally, the effectiveness of the proposed method was evaluated using three metrics:

1. Maximum interaction force component: $\max∥F_{ei}∥,i=x,y,z$;
2. Relative root mean square error(RRMSE): $RRMSE\_i=\sqrt{\frac{1}{T}\sum_{t=0}^{T}\frac{{\left(F_{ei}-F_{di}\right)}^{2}}{F_{di}^{2}}},i=x,y,z$, calculated over the trial time *T*;
3. Force constraint violation rate: $\eta =\frac{T_{h}}{T_{total}}$, where $T_{h}$ represents the duration during which the interaction forces exceed the maximum constraint force, and $T_{total}$ represents the total task duration.

Metrics for each method are listed in Table 2. The maximum force component decreased from 68.6 mN with the TIA method to 20.44 mN using the proposed method. This indicates that the proposed method can significantly reduce hazardous interaction forces. The RRMSE and η values for the proposed method are significantly lower than those of the control methods, indicating a reduced risk of unsafe interaction forces. These results suggest that the master–slave robot system, employing the proposed method, effectively enhances interaction force management in intraocular microsurgery.

## 6. Discussion

Compared to the TIA method and the TIA method with haptic feedback, the proposed framework takes slightly longer for membrane peeling but significantly reduces the peeling force, thereby improving surgical safety. Reference [7] reported six clinical membrane peeling procedures performed using the teleoperated robotic system, with a median completion time of 295 s and an interquartile range (IQR) of 140 s. In comparison, the proposed framework in this study requires approximately 344 s for membrane peeling, indicating that the time expenditure is acceptable.

Existing studies on force control for membrane peeling typically focus on force component control. This approach precisely controls interaction forces in each direction, providing flexibility and specificity. However, force component control can lead to excessive resultant forces. This may cause premature tearing of the membrane. In contrast, force norm control enhances safety by regulating interaction force magnitude. However, it may hinder membrane peeling due to forces in other directions. Further evaluation of force component control and force norm control is required. This will help identify the optimal force control strategy for membrane peeling.

The tape used in the experiment differs significantly from ERM and ILM in morphology and biomechanical properties. Therefore, we plan to conduct experiments on real eyeballs in the future to further validate the stability, reliability, and generalizability of the proposed framework.

## 7. Conclusions

This paper focuses on interaction force constraint in retinal surgery assisted by a teleoperated robotic system. A haptic shared control framework based on a force-constrained supervisory controller has been proposed. When the interaction forces on the tool tip approach the maximum limit, the force supervisory controller minimally modifies the user’s commands to maintain interaction forces within safe ranges. The proposed framework has been validated through simulation experiments and simulated membrane peeling experiments. The results have shown that the proposed framework significantly reduces the rate of force constraint violation for teleoperation without the robot’s dynamic model and the safety margin. Although the peeling time increases with the introduction of proposed framework, excessive interaction forces are significantly reduced. The proposed framework prevents unnecessary damage to the human eye during the retinal surgery. In future work, we will further evaluate the effects of force component control and force norm control in biological membrane peeling to validate the feasibility and generalizability of the proposed framework.

## Figures and Tables

**Table 1:** Parameter settings for different force control methods.

Method	Controller	Values of Parameters
Proposed method	$\begin{array}{c} u_{pos}=k_{p}\left(z_{d}-z\right),\\ u=\arg\min\frac{1}{2}{∥u^{*}-u_{pos}∥}^{2},\\ s.t.\begin{array}{cc} & K_{e}u^{*}\leq \alpha_{e}\left(f_{d}-f_{e}\right) \end{array}. \end{array}$	$k_{p}=1,\alpha_{e}=70$
Force/position hybrid method [11]	$\begin{array}{c} u_{pos}=k_{p}\left(z_{d}-z\right),\\ u=\left(1-\delta \right)u_{pos}+\delta \left(\frac{k_{f}}{Ke}\left(f_{d}-f\right)\right). \end{array}$	$\begin{array}{c} k_{p}=1,k_{f}=70,\\ \delta =\left\{\begin{array}{c} 0,f<f_{d}\\ 1,others \end{array}\right.. \end{array}$
Impedance control [17]	$u=f_{d}-f-k\left(z-z_{d}\right)-b\left(\dot{z}-{\dot{z}}_{d}\right).$	k=1,b=30.

**Table 2:** Experimental metrics using different methods.

Method	TIA	TIA with Haptic Feedback	Proposed Method
$\max∥F_{ei}∥$ (mN)	68.60	58.53	20.44
RRMSE_X	0.4995	0.1625	None ^1^
RRMSE_Y	1.0541	0.7205	0.0085
RRMSE_Z	0.1174	None ^1^	0.0081
$T_{total}$ (s)	23.61	97.71	343.8
$T_{h}$ (s)	20.1	79.77	5.91
η	85.13%	81.64%	1.72%

^1^ The interaction force in this direction does not exceed the maximum constraint value in the experiment.

## Data Availability

Data are contained within the article.
